# Effect of Customized Insoles on Gait in Post-Stroke Hemiparetic Individuals: A Randomized Controlled Trial

**DOI:** 10.3390/biology10111187

**Published:** 2021-11-15

**Authors:** Jie Wang, Lei Qiao, Long Yu, Yanmin Wang, Redha Taiar, Ying Zhang, Weijie Fu

**Affiliations:** 1 Department of Rehabilitation, Shanghai Xuhui Central Hospital, Shanghai 200030, China; wangjie922@126.com (J.W.); 13370217879@163.com (L.Q.); yulong6661112@163.com (L.Y.); razorback_1222@126.com (Y.W.); 2 School of Kinesiology, Shanghai University of Sport, Shanghai 200438, China; 3 MATIM, Department of Sport Science, Université de Reims Champagne Ardenne, 51100 Reims, France; rtaiar@chu-reims.fr

**Keywords:** Tinetti Gait Scale (TGS), customized insoles, stroke rehabilitation, gait

## Abstract

**Simple Summary:**

Stroke patients commonly have different lower extremity biomechanical abnormalities that severely affect walking after damage to normal neural pathways, however, little attention has been paid to them, and current gait rehabilitation techniques have made limited efforts to provide patients with consistent, stable, and effective correction when walking. In the present study, we investigated whether customized insoles could improve gait performance in hemiplegic stroke patients, and the results showed that customized insoles could be a valid intervention that targets residual hemiplegic gait after stroke, thereby enhancing walking function and improving the quality of life of the patients.

**Abstract:**

**Background:** Insoles have been widely applied to many diseases, but stroke involves complex problems and there is a paucity of research on the application of insoles in stroke patients. **Aim:** To evaluate the effect of customized insoles on gait in patients with hemiplegia. **Design:** A randomized controlled trial. **Setting:** Rehabilitation department of a hospital. **Population:** A total of 50 stroke patients were randomized into an experimental group (n = 25) or a control group (n = 25). **Methods:** Both groups received conventional gait training, which was conducted five times a week, every 40 min for four weeks and patients in the experimental group were required to wear customized insoles for at least 1 h per day for four weeks. The primary outcome measure was the Tinetti Gait Scale (TGS) and the secondary outcome measures were the plantar pressure test, 6-min walking test (6MWT), lower extremity Fugl–Meyer assessment (FMA-LE), Berg Balance Scale (BBS), and the modified Barthel index (MBI). **Results:** Compared to the control group, there were significant increases in the experimental group after four weeks (*p* = 0.014) and at the four week follow-up (*p* = 0.001) in the change in TGS, weight-bearing on the involved side (*p* = 0.012) or forefoot (*p* = 0.028) when standing, weight-bearing on the involved side (*p* = 0.01 6) or forefoot (*p* = 0.043) when walking, early stance phase (*p* = 0.023) and mid stance phase (*p* = 0.013) on the involved side, FMA-LE (*p* = 0.029), BBS (*p* = 0.005), and MBI (*p* = 0.009), but there were no differences in the late stance phase (*p* = 0.472) on the involved side when walking or in the 6MWT (*p* = 0.069). **Conclusions:** Customized insoles had great efficacy in enhancing gait performance in stroke patients.

## 1. Introduction

Approximately 80% of stroke patients suffer from hemiplegic gait [1]. Hemiplegic gait includes a prolonged swing phase, shortened stance phase, increased asymmetry in time and space on the involved side [2], and the lower extremity often presents as hip external rotation, knee hyperextension, foot drop, and varus [3]. Moreover, the increasing incidence of falls caused by hemiplegia greatly lowers the patients’ quality of life and causes an economic burden for health and social services [4].

A wide range of strategies in conventional gait therapy have been developed [5] such as manipulation provided by physical therapists and technological approaches including functional electric stimulation (FES) [6] or treadmill training [7]. Stroke patients who receive conventional gait therapy have to stick with certain therapists or specific locations. Therefore, losing access to rehabilitation facilities or therapists may result in a relapse of the gait pattern, which should be particularly noticeable [8]. Previous studies have reported that hemiplegic patients often experienced changes in plantar pressure due to biomechanical abnormalities of the lower extremities. These abnormalities altered the closed chain movement of the upper segment of the body, eventually aggravating abnormal gait [9,10,11]. However, few studies have focused on rehabilitation techniques based on abnormal plantar pressure for hemiplegic patients.

In recent years, treatments with corrective insoles have been widely applied to many conditions such as flatfoot, plantar fasciitis, diabetic foot, posterior tibial tendonitis, lower back pain, and others [12,13,14,15,16]. Corrective insoles can change the pressure distribution on the sole to provide an appropriate base of support, maintain correct positioning of the foot to limit or facilitate the movement of the lower limbs as well as enhance shock absorption and stability to alleviate pain or other specific pathologies while standing or walking [17]. Previous research has demonstrated that lateral-wedged and heel-lift insoles had a positive effect by increasing the weight-bearing of the hemiplegic side and bilateral symmetry during standing, but these effects were not felt during ambulation [18,19]. Kusumoto et al. confirmed that the insoles combined with a metatarsal pad promoted static and dynamic balance in patients with cerebral palsy [20]. Based on the previously mentioned studies, it was found that the insoles had only one additional modification that had been applied for the correction of lower limb kinematic and kinetic abnormalities in most studies on hemiplegic gait [21,22]. In fact, patients with hemiplegia usually had variable abnormal gait performances, which were caused by different foot structures and movement defects such as a collapsed transverse arch, spastic clawing of toes, restricted ankle dorsiflexion, and plantarflexion [23]. These problems often could not be solved with a single additional modification of insoles, findings that were not conducive to the patient’s gait smoothness and stability.

As a type of corrective insoles, customized insoles are individually and specifically applied to biomechanical deformities by adding various pads (e.g., inversion ramp pad, forefoot pad, metatarsal dome pad, hallux valgus pad, etc.) to prefabricated insoles, and each pad has its own function [22]. This technique takes the personal biomechanical problems of each patient into account. Additionally, one of the prominent advantages of customized insoles is that they can be further modified after production, unlike 3D-printed insoles, which cannot be changed once the product is formed [24]. Therefore, the purpose of the present study was to evaluate whether wearing customized insoles could improve gait in stroke patients and the impact of this technique on plantar pressure distribution, lower limb motor function, balance function, daily life ability, and walking endurance.

## 2. Materials and Methods

### 2.1. Study Design

We designed a single-blind, randomized clinical trial to examine the effects of customized insoles on the gait of patients with hemiplegia. Eligible patients were randomized into ‘conventional gait training + customized insoles’ (Group A) or ‘conventional training’ (Group B) at a 1:1 ratio using a computer-generated random table. We put trial instructions and groupings in sealed envelopes and the patients were randomly assigned in order. The study was approved by the Ethics Committee of the Central Hospital of Xuhui District, Shanghai (No. 2019-54).

Each patient was evaluated by the same assessor who was unaware of the group assignment. The primary outcome measures were conducted three times (baseline [T_0_], four weeks from baseline [T_1_] and four weeks after completion of the intervention [T_2_]) and the secondary outcomes were assessed twice (baseline [T_0_] and four weeks from baseline [T_1_]).

### 2.2. Patients

From July 2019 to July 2020, a total of 50 patients were recruited from the outpatient department and ward of the rehabilitation department of our hospital. Patient recruitment included the following steps: (1) a patient’s attending doctor was required to be acquainted with the inclusion and exclusion criteria as well as the screening of potential participants and recommending them to the primary researcher; (2) the patients were then judged on their clinical characteristics; (3) the primary researcher explained the trial aims to each potential patient and discussed the rehabilitation objectives with them; and (4) the patients or their relatives were required to sign informed consent forms.

Inclusion criteria for subjects were: (1) met the diagnostic criteria for cerebral infarction or cerebral hemorrhage; (2) unilateral limb paralysis from the first stroke; (3) time of onset: 1–12 months after suffering a stroke; (4) aged 40–80 years; (5) involved lower limb motor function ≥Brunnstrom grade III; (6) ability to walk at least 10 m with or without auxiliary tools; (7) no other diseases or complications that might affect rehabilitation training; and (8) stable vital signs.

Exclusion criteria were: (1) a history of any other additional diseases that could influence ambulation; (2) diabetic foot and peripheral neuropathy; (3) MMSE <17 points; (4) severe communication impairment; and (5) other factors that might prevent participation in the trial.

### 2.3. Intervention

All patients underwent conventional gait training. During ambulation, experienced therapists, who had passed the health professional qualification appraisal, performed manipulations to help patients suppress excessive muscle tension, stimulate muscle activity, and promote normal movement patterns. In addition, patients received instructions about weight-shifting, involved limb weight-bearing, balance training, and various intensive exercises as functional activities (such as standing up from a chair, turning around, crossing obstacles, and so on). Both groups received 40-min conventional gait training once a day, 5 times a week, for 4 weeks. Only patients in the experimental group were required to wear insoles for a minimum of 1 h every day and recommended that they continued wearing them at the 4-week follow-up after the completion of treatment, but it was not mandatory.

#### Customization Processes of Insoles

**Figure 1:**
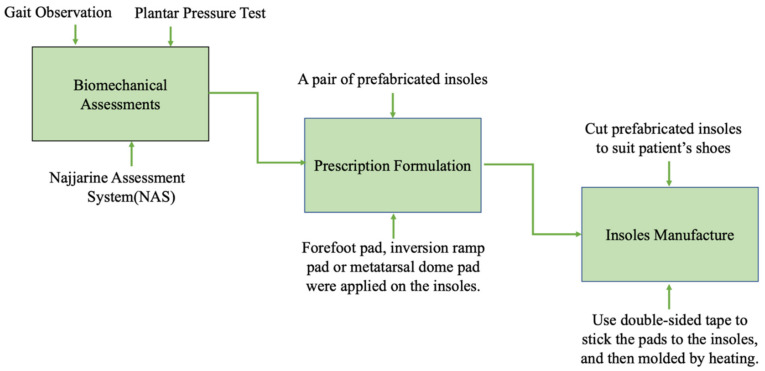
Customization process of the insoles.

The process included relevant biomechanical assessments, prescription formulation, and the local manufacture of the insoles (Figure 1).

*Step 1:* The assessments mainly consisted of gait observation, plantar pressure test (F-Scan^®^, Foot Analyzer ver. 2.0.1,Techstrom, Korea), and the use of the Najjarine assessment system (NAS) [25]. Gait observation required the therapist to observe the condition of each segment of the movement chain from the front, back, and side aspects, respectively, especially the movement of the involved lower limb and foot [26]. During the plantar pressure test, a patient stood naturally on the electronic plantar pressure plate and remained while static data were collected, then stepped over the plate to collect dynamic data [27]. Gait observation and the plantar pressure test can reveal neuromuscular abnormalities of the foot and ankle after central nervous system injury. With NAS, leg length, forefoot to rearfoot position, and the calcaneal angle when standing, which reflects the patients’ foot structure and the biomechanical status of lower limbs, can be determined.

**Figure 2:**
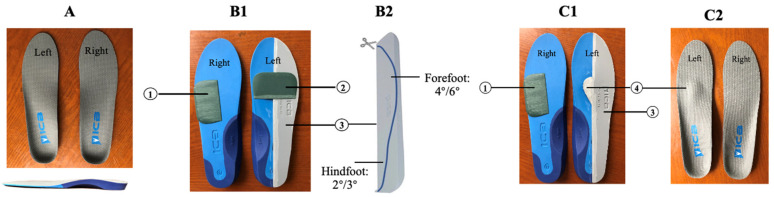
Conventional prescriptions for hemiplegia (left-sided hemiplegia). Note: (**A**) demonstrated a pair of prefabricated insoles with a 5° lateral wedge and arch support. (**B1**–**C2**) demonstrated two typical prescriptions for hemiplegia: (1) forefoot pad (2° or 4°), which was stuck on the lateral of the unparetic forefoot to increase the stability of the unaffected foot; (2) forefoot pad (2° or 4°), which was stuck on the paretic forefoot in order to promote ankle dorsiflexion; (3) inversion ramp pad (4° or 6°), which was stuck on the lateral of the paretic foot. The whole pad was trimmed along the line to reach forefoot 4°-hindfoot 2° or forefoot 6°-hindfoot 3°, and its medial edge was polished into a slope to promote the paretic foot’s pronation movement; and (4) metatarsal dome pad, which was stuck slightly posterior to the metatarsal heads in order to alleviate paretic forefoot plantar pressure on the paretic side. All of the above pads were stuck to the plantar surface of the insole.

*Step 2:* Based on the results of the above assessments, an individualized prescription of insoles was designed for each stroke patient with hemiplegia. A pair of prefabricated insoles, with a 5° lateral wedge, and an arch support made from high-density ethyl vinyl acetate (ICB Dual Density Orthotics, Jiangsu Suyun Medical Equipment Co. Ltd., Lianyungang, China), were distributed to each patient in the experimental group. Typically, we added a 2° or 4° forefoot pad on the paretic forefoot to promote ankle dorsiflexion, a 4° or 6° inversion ramp pad to promote the paretic foot’s pronation movement, and a metatarsal dome pad to alleviate paretic forefoot plantar pressure on the involved side. In addition, a 4° forefoot pad was added on the lateral of the non-paretic forefoot to increase the stability of the uninvolved foot. All the above pads were attached to the plantar surface of the insoles with double-sided tape (Figure 2).

*Step 3*: The producers chose and cut appropriate-size prefabricated insoles to suit the patients’ shoes and all pads were pre-cut and attached to the insole. After the insoles were fabricated, the patient tried them on and appropriate adjustments and corrections were performed if necessary. Finally, the insoles were molded, specifically by heating them for 40 s with a heat gun and then shaped by a qualified physiotherapist to maintain the subtalar joint in a neutral standing position [28]. Furthermore, some modifications could be made according to the patient’s condition at a later time.

All of the above steps were performed by experienced qualified therapists.

### 2.4. Outcome Measures

TGS measurement was our primary indicator [29], which has good interrater reliability (r = 0.80–0.89) [30] and was used to evaluate the patient’s gait. It consisted of six items including two items related to coordinated gait components, five items related to compensation strategies, and one item related to temporal aspects of gait. TGS ranged from 0 to 12 points, representing most deviations from normal.

#### Secondary Outcomes

- Plantar pressure test (%) [31]: Items included weight-bearing of the involved foot (normal: 50%) and the involved forefoot (normal: 27.5%) during static standing, weight-bearing of the involved foot (normal: 50%), and the involved forefoot (normal: 35%) during walking, and gait cycle percentage (early stance phase, normal: 20%; mid-stance phase, normal: 40%; and late stance phase, normal: 20%).
- 6MWT [32]: This was carried out indoors along a long, flat, straight enclosed corridor. The length of the walking track was 30 m and subjects walked as fast as they could for 6 min, and the walking distance was measured.
- FMA-LE [33]: This is a reliable tool for assessing stroke recovery (ICC = 0.83–0.95) [34]. There are 17 items in this assessment, of which two items relate to reflex activity, 11 items to synergistic movements, and three items to coordination. The scoring of each item was based on a sequential score of three points (0, unable to complete; 1, partially completed; 2, completed), except for the two reflection items.
- BBS [35]: This exhibits good interrater reliability (ICC = 0.95–0.98) [36] and is a list of 14 items, and each item comprised of a 5-point ordinal scale from 0 to 4; 0 represents the lowest level of function and 4 the highest level of function.
- BI assessment [37]: This exhibits good interrater reliability (ICC = 0.94–0.98) [38] and covers 10 domains of functioning (activities): bowel and bladder control as well as assistance with grooming, toilet use, feeding, transfer, walking, dressing, climbing stairs, and bathing. Each activity has five dependency levels ranging from 0 (unable to perform) to 5, 10, or 15 (completely independent).

All assessments were conducted when patients wore shoes with insoles, except for lower limb function and plantar pressure.

### 2.5. Statistical Analysis

Data analysis was performed using SPSS Statistics (SPSS 20.0, SPSS Inc., Chicago, IL, USA). Patients’ demographic and clinical characteristics including age, gender, course and classification of stroke, involved side (left/right) and the Brunnstrom stage are given by the number of cases (%) for categorical data and mean (SD) for continuous variables in Table 1. Shapiro–Francia normality test analysis was performed before our statistical analysis [39]. TGS was described as the number of cases (%) and the plantar pressure test, FMA-LE assessment, and BBS, which did not conform to a normal distribution as well as discontinuous variables such as the BI assessment are presented as the mean (95% confidence interval (CI). Continuous variables such as the 6MWT with a normal distribution are reported as the mean ± SD.

A Chi-squared test was conducted for comparison of changes in the TGS. The Wilcoxon rank sum test was used to analyze differences between the two groups and the Wilcoxon signed-rank test was employed within the groups for discontinuous variables such as TGS and BI assessments. Based on the assumption of normal distribution and homogeneity of variance, we conducted an independent sample *t*-test to analyze the difference in the change in 6MWT between the two groups. Continuous variables such as the change in the plantar pressure test, FMA-LE assessment, and BBS did not exhibit a normal distribution and Wilcoxon signed-rank test was used within the groups and Wilcoxon rank sum test between groups. The significant level was set as α = 0.05.

## 3. Results

**Figure 3:**
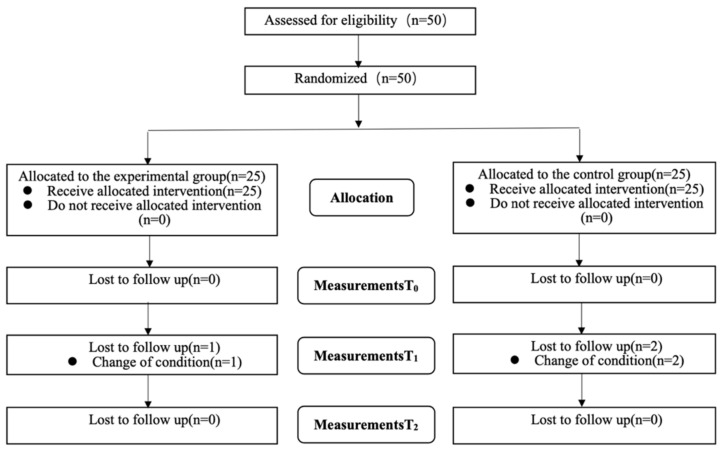
Flowchart (T_0_: baseline; T_1_: four weeks from baseline; T_2_: four weeks after completion of the intervention).

As shown in Figure 3, a total of 50 patients met the inclusion criteria and were enrolled in the study and 47 patients completed the experimental procedures and follow-up measurement data. Analysis was by intention to treat and three patients (one in the experimental group and two in the control group) dropped out and their missing data were filled with the most recent data. All were included in the statistical analysis and there was no significant difference between the experimental group and the control group for baseline demographic data and all outcome measures (Table 1). No serious advents were observed.

After four weeks of intervention, the change in the TGS in the experimental group was significantly different from the control group (*p* = 0.014). Furthermore, a comparison of the changes in the TGS after four weeks follow-up also showed that the experimental group was higher than the control group (*p* = 0.001) (Table 2 and Table 3). After four weeks of intervention, changes in weight-bearing on the involved side (*p* = 0.012) and forefoot (*p* = 0.028) when standing between groups exhibited a significant difference, and statistically significant differences were found in the change of weight bearing on the involved side (*p* = 0.016) and forefoot (*p* = 0.043) when walking. The change in the early stance phase (*p* = 0.023) and mid stance phase (*p* = 0.013) on the involved side exhibited statistical differences between groups, but no statistical difference in the change of the late stance phase on the involved side (*p* = 0.472). In addition, there were significant differences in the change in FMA-LE between the two groups (*p* = 0.029), BBS (*p* = 0.005) and BI (*p* = 0.009), while the 6MWT exhibited no significant differences between the two groups (*p* = 0.069) (Table 4).

## 4. Discussion

The study results revealed that patients in the experimental group exhibited significantly improved gait performance compared with the control group, after the treatment period and at the 4-week follow-ups. In addition, after four week treatment of the intervention group, weight-bearing on the involved foot or forefoot was increased regardless of whether the patient was standing or walking. The early stance phase was prolonged and the mid-stance phase was shortened. Furthermore, the use of customized insoles was more effective in enhancing lower limb motor function, balance, and activities of daily life, except for the patients’ walking endurance.

Studies have shown that the joint kinematics and temporospatial features of hemiplegia patients are different from those in healthy people during the stance and swing phase [40,41]. In the early stance phase, the subtalar joint is in a supination position and the limited dorsiflexion of the ankle causes the involved foot to tend to land on the lateral heel, forefoot, or flat-foot at initial contact, which would further impede knee flexion [42]. In the mid-stance phase, due to limited ankle dorsiflexion, forward progression of the leg is not allowed; thus, patients with hemiparesis often present with compensatory hip flexion and trunk forward leaning [43]. When the involved side was in the prolonged swing phase [42], the uninvolved side was exactly in the middle and late phase of standing. The dynamic transformation under this condition was that the center of gravity should be moved to the involved side, while the uninvolved side would be moved forward simultaneously, which is a necessary requirement for patient balance. We found that the center of gravity moved back-and-forth, inward-and-outward not only on the involved side, but also on the uninvolved side in the forefoot and midfoot. Therefore, the uninvolved foot is also in an unstable state, which is very worthy of attention and the same stated phenomenon is consistent with the findings in the research by Merying et al. [44,45].

The methods to modify insoles in our study were to add different combinations of pads to a pair of full-length prefabricated insoles. The inversion ramp on the lateral region of the involved foot encouraged the paretic foot to move from supination at heel contact into maximum pronation by the time the forefoot had contacted the floor. The inversion ramp also re-established the heel as an appropriate base of support, promoted forward motion of the tibia, and restored the rocker action of the ankle, thus assisting in propulsion [46]. Moreover, we modified the insole with a forefoot pad under the paretic forefoot to induce ankle dorsiflexion in the mid-stance phase. Simultaneously, knee flexion should also be improved along the human movement chain. A metatarsal dome was added, slightly posterior to metatarsal heads, to mend claw toe, alleviating the hypertonicity and abnormal pressure distribution of the forefoot. As above-mentioned, there was instability in the uninvolved foot and previous literature has reported that lateral pads could alleviate instability in the elderly during walking [47]. With this in mind, we added a lateral forefoot pad to the uninvolved forefoot to enhance its stability, consequently improving the balance of the individual and making the gait smooth.

The results of the TGS analysis indicated improvement in gait performance in the experimental group. The advantages of the customized insoles were: (1) they helped to maintain good foot posture by providing an appropriate base of support, thus reducing compensatory movement; and (2) they enhanced the stability of the uninvolved side [48]. Indeed, patients with customized insoles exhibited a smoother gait after 4-weeks of intervention, and the daily logs of patients showed an average of 2.87 h spent wearing insoles per day during the intervention period. Surprisingly, most patients in the experimental group were willing to voluntarily wear the customized insoles for an average of 2.59 h per day during the 4-week follow-up period. Thus, wearing customized insoles was a constant and convenient treatment for stroke patients compared to conventional gait therapy, which clearly had restrictions on locations and access to professionals.

A major reason for the success of the customized insoles was that they were designed to redistribute the load of the foot, resulting in a better distribution of plantar pressure. The inversion ramp on the lateral region increased plantar foot contact area during the phase of initial heel contact from flat foot to floor. As a consequence, the results of the plantar pressure test showed that wearing customized insoles promoted weight-bearing on the involved foot or forefoot during a movement of static standing or dynamic walking. Certainly, the early stance phase was also prolonged for the paretic foot. In addition, the forefoot pad induced active dorsiflexion of the involved foot and the metatarsal dome relieved the excessive pressure of the toe, which made the center of gravity move forward more easily. Consequently, this shortened the mid-stance phase of the involved side.

Based on the above reason, the customized insoles also promoted the motor function of the lower limbs for hemiplegia patients. Thus, the results of the present study demonstrated that the experimental group had greater benefits than the control group in the case of the FMA-LE assessment, a finding which is in agreement with Li’s research [49].

The intervention group further gained greater results in comparison to the control group when we investigated the changes associated with the BBS and BI scores after four weeks, which illustrated that the insoles were effective in improving the balance of stroke patients as well as contributing to an overall improvement in daily life. Clinically, the customized insole re-established a proper and steady foundation for hemiplegic patients during the gait cycle, thus improving a patient’s balance function. Studies have determined that due to the improvements associated with lower limb and balance function, the ability of toileting, transferring, ambulating, and stair climbing were also enhanced [50,51].

The 6MWT is a long walking task, which was designed to measure the endurance of the patient and is believed to correlate well with community activities. In this assessment, the patient was asked to walk as far as possible in 6 min. Stroke patients already had a slower gait speed [52] and previous studies have shown that 6MWT is a poor predictor for people with a slower walking speed [5]. Therefore, our study found that there was no significant difference in the performances of the 6MWT between the experimental and control groups after four weeks of the intervention.

### Study Limitations

First, the primary observed indicator used was scale, so three-dimensional gait analysis may be considered to quantify objectively gait performance. Second, the sample size of this study was relatively small, which may cause uncertainty; more patients will be included in the cohort to verify the efficacy of customized insoles in future studies. Finally, in subsequent investigations, positive control studies can be used such as a comparison with ankle-foot orthoses, which may have more clinical significance in evaluating the effect of customized insoles. A stratified study should be carried out according to different levels of lower extremity motor function of patients to determine which level or levels the customized insoles are more suitable, and to provide further evidence for the clinical application of insoles in stroke hemiplegia.

## 5. Conclusions

Customized insoles were effective as a type of orthotic treatment designed to improve the gait performance of patients with hemiplegia. The benefits included improved gait cycle, increased weight-bearing on the involved side when static standing and dynamic walking, better motor function of lower limbs, balance, and overall abilities in daily life.

## Figures and Tables

**Table 1:** Patient characteristics at baseline (Group A: experimental group; Group B: control group).

Characteristic	Group A	Group B	*p*-Value
Participants, n	25	25	
Age (years), median (IQR)	56.00 (49.50 to 66.50)	60.00 (54.00 to 65.00)	0.303
Duration of stroke (days), mean (SD)	130.36 (64.87)	123.08 (54.06)	0.668
Male, n (%)	19 (76%)	18 (72%)	0.747
Classification, n (%)			
Cerebral infarction	13 (52%)	16 (64%)	0.390
Cerebral hemorrhage	12 (48%)	9 (36%)	
Affected body side, n (%)			
Left	17 (68%)	18 (72%)	0.758
Right	8 (34%)	7 (28%)	
Brunnstrom, n (%)			
III	5 (20%)	4 (16%)	0.762
IV	16 (64%)	15 (60%)	
V	4 (16%)	6 (24%)	

**Table 2:** Results of the Tinetti Gait Scale (TGS).

TGS Score, n (%)	T_0_		T_1_		T_2_	
	Group A (n = 25)	Group B (n = 25)	Group A (n = 25)	Group B (n = 25)	Group A (n = 25)	Group B (n = 25)
4	1 (4%)	1 (4%)	1 (4%)	0	1 (4%)	0
5	10 (40%)	4 (16%)	1 (4%)	0	0	1 (4%)
6	4 (16%)	9 (36%)	2 (8%)	6 (24%)	0	4 (16%)
7	5 (20%)	7 (28%)	1 (4%)	6 (24%)	3 (12%)	8 (32%)
8	4 (16%)	3 (12%)	9 (36%)	7 (28%)	13 (52%)	9 (36%)
9	1 (4%)	1 (4%)	8 (32%)	6 (24%)	7 (28%)	3 (12%)
10			3 (12%)	0	1 (4%)	0

Note: Group A: experimental group; Group B: control group. T_0_: baseline measurement; T_1_: four weeks from baseline; T_2_: four weeks after completion of the intervention.

**Table 3:** Changes in the Tinetti Gait Scale (TGS).

Change in TGS, n (%)	T_1_–T_0_		T_2_–T_0_	
	Group A (n = 25)	Group B (n = 25)	Group A (n = 25)	Group B (n = 25)
0	3 (12%)	5 (20%)	1 (4%)	4 (16%)
1	5 (20%)	14 (56%)	3 (12%)	1 (4%)
2	8 (32%)	4 (16%)	2 (8%)	13 (52%)
3	9 (36%)	2 (8%)	10 (40%)	6 (24%)
4			9 (36%)	1 (4%)
*p*-value	0.014		0.001	

Note: Group A: experimental group; Group B: control group. T_0_: baseline measurement; T_1_: four weeks from baseline; T_2_: four weeks after completion of the intervention. The *p*-values refer to differences of the change of the outcome measures between the two groups, *p* < 0.05.

**Table 4:** Results of the secondary outcomes.

	T_1_–T_0_		*p*-Value
	Group A	Group B	
Weight-bearing on the affected side, standing, %, mean (95% CI)	3.46 (2.37 to 4.55)	1.49 (0.48 to 2.49)	0.012
Weight-bearing on the forefoot of the affected side, standing, %, mean (95% CI)	2.73 (1.67 to 3.79)	1.49 (0.65 to 2.34)	0.028
Weight-bearing on the affected side, walking, %, mean (95% CI)	3.15 (1.53 to 4.77)	1.33 (0.49 to 2.17)	0.016
Weight-bearing on the forefoot, walking, %, mean (95% CI)	4.06 (2.14 to 5.99)	1.93 (0.67 to 3.20)	0.043
Early stance phase, %, mean (95% CI)	5.00 (3.21 to 6.79)	2.68 (1.86 to 3.50)	0.023
Mid stance phase, %, mean (95% CI)	−5.68 (−8.29 to −3.07)	−1.36(−3.10 to 0.38)	0.013
Late stance phase, %, mean (95% CI)	0.68 (−2.28 to 3.64)	−1.32 (−3.23 to 0.59)	0.472
6MWT, mean (SD)	64.68 (32.12)	47.88 (31.67)	0.069
FMA-L, mean (95% CI)	7.00 (5.97 to 8.03)	5.48 (4.52 to 6.44)	0.029
BBS, mean (SD)	6.28 (2.99)	4.04 (2.35)	0.005
BI, mean (95% CI)	12.40 (10.24 to 14.56)	9.00 (7.31 to 10.69)	0.009

Note: Group A: experimental group; Group B: control group. 6MWT: 6-min walking test; FMA-LE: lower extremity Fugl–Meyer assessment; BBS: Berg Balance Scale; BI: Barthel index. T_0_: baseline measurement; T_1_: four weeks from baseline; *p*-values refer to the differences in the change in the outcome measures between the two groups, *p* < 0.05.

## Data Availability

Data related to this study are available from the corresponding author. The data cannot be made public because of privacy or ethical restrictions.
